# Measuring resilience by cognitive diagnosis models and its prediction of 6-month quality of life in Be Resilient to Breast Cancer (BRBC)

**DOI:** 10.3389/fpsyt.2023.1102258

**Published:** 2023-02-16

**Authors:** Mu Zi Liang, Peng Chen, M. Tish Knobf, Alex Molassiotis, Ying Tang, Guang Yun Hu, Zhe Sun, Yuan Liang Yu, Zeng Jie Ye

**Affiliations:** ^1^Guangdong Academy of Population Development, Guangzhou, Guangdong, China; ^2^Basic Medical School, Guizhou University of Traditional Chinese Medicine, Guiyang, China; ^3^School of Nursing, Yale University, Orange, CT, United States; ^4^College of Arts, Humanities and Education, University of Derby, Derby, United Kingdom; ^5^Institute of Tumor, Guangzhou University of Chinese Medicine, Guangzhou, China; ^6^School of Nursing, Army Medical University, Chongqing, China; ^7^The First Affiliated Hospital, Guangzhou University of Chinese Medicine, Guangzhou, Guangdong, China; ^8^Mental Health Education and Counseling Center, South China University of Technology, Guangzhou, Guangdong, China; ^9^School of Nursing, Guangzhou University of Chinese Medicine, Guangzhou, Guangdong, China

**Keywords:** cognitive diagnosis models (CDMs), cognitive diagnostic probabilities, resilience, 6-month quality of life (QoL), breast cancer, prediction model, multicenter cohorts

## Abstract

**Objective:**

The application of advanced Cognitive Diagnosis Models (CDMs) in the Patient Reported Outcome (PRO) is limited due to its complex statistics. This study was designed to measure resilience using CDMs and its prediction of 6-month Quality of Life (QoL) in breast cancer.

**Methods:**

A total of 492 patients were longitudinally enrolled from Be Resilient to Breast Cancer (BRBC) and administered with 10-item Resilience Scale Specific to Cancer (RS-SC-10) and Functional Assessment of Cancer Therapy-Breast (FACT-B). Generalized Deterministic Input, Noisy “And” Gate (G-DINA) was performed to measure cognitive diagnostic probabilities (CDPs) of resilience. Integrated Discrimination Improvement (IDI) and Net Reclassification Improvement (NRI) were utilized to estimate the incremental prediction value of cognitive diagnostic probabilities over total score.

**Results:**

CDPs of resilience improved prediction of 6-month QoL above conventional total score. AUC increased from 82.6–88.8% to 95.2–96.5% in four cohorts (all *P* < 0.001). The NRI ranged from 15.13 to 54.01% and IDI ranged from 24.69 to 47.55% (all *P* < 0.001).

**Conclusion:**

CDPs of resilience contribute to a more accurate prediction of 6-month QoL above conventional total score. CDMs could help optimize Patient Reported Outcomes (PROs) measurement in breast cancer.

## Introduction

Breast cancer accounts for 24.5% of all cancer cases in women and 2.3 million new cases are identified worldwide in 2020 (1). Among which, 18.3% of all breast cancer cases (about 420 thousand) occur in China. In addition, with advances in early detection and new therapies of breast cancer, 5-year survival has increased to near 90% and breast cancer is now treated as a chronic disease (1, 2). However, breast cancer survivors still suffer from many psychosocial burdens including stigma, depression, anxiety, fear of cancer recurrence, etc., which will result in reduced Quality of Life (QoL) (3, 4). Different from physical symptoms (i.e., lymphedema), psychosocial burdens cannot be measured by objective indicators and Patient Reported Outcomes (PROs) are developed to improve the detection of the patients’ subjective experience (5). For example, Self-Rating Depression Scale (SDS), the Center for Epidemiologic Studies Depression Scale (CES-D) and the Beck Depression Inventory (BDI) have been well developed to assess the severity of the depressive disorder (6–8). However, many existing PROs instruments are developed based on classical test theory (CTT) and focus on the accurate estimation of the symptom severity (i.e., depression, fatigue, etc.), which cannot provide detailed information at item-level. For example, most depression inventories are unidimensional and total scores are calculated to estimate the severity of the depressive symptoms with several cutoffs as reference. This procedure is straightforward but item-level information (specific symptom) is neglected, which is not consistent with definition in 10th revision of the International Classification of Diseases (ICD-10) and 5th edition of the Diagnostic and Statistical Manual of Mental Disorders (DSM-5) (9, 10).

Cognitive Diagnosis Models (CDMs), or diagnostic classification models (DCMs), are state of art psychometric models, which have been developed to classify participants into latent classes with unique profiles of attributes (11). Compared with CTT or item response theory (IRT), the CDMs provide an alternative psychometric framework for test development and score reporting. Due to the complex statistics in CDMs, most CDMs-related articles are published in the field of education measurement. However, multidiscipline researchers have been recently aware of their usefulness in PROs assessments for symptom profiles at item-level, which will be helpful to screening or intervention (12–14). Further, compared with factor analysis in CCT, CDMs allow latent attributes/symptom criteria to interact resulting in a more flexible psychometric model. Therefore, from a different perspective, the current study was designed to provide more information for the screening and monitoring of resilience as an example under the framework of CDMs. Based on the data from our previous Be Resilient to Breast Cancer (15–18), the current study was designed to compare the prediction ability of resilience to 6-month Quality of Life (QoL), between total score based on CTT and cognitive diagnostic probabilities (CDPs) based on CDMs. We hypothesized that: (1) CDMs could well extract diagnostic details about patients’ resilience strengths and weaknesses, (2) CDPs of resilience could offer incremental predictive value above conventional total score.

## Materials and methods

### Patients and data collection

**FIGURE 1:**
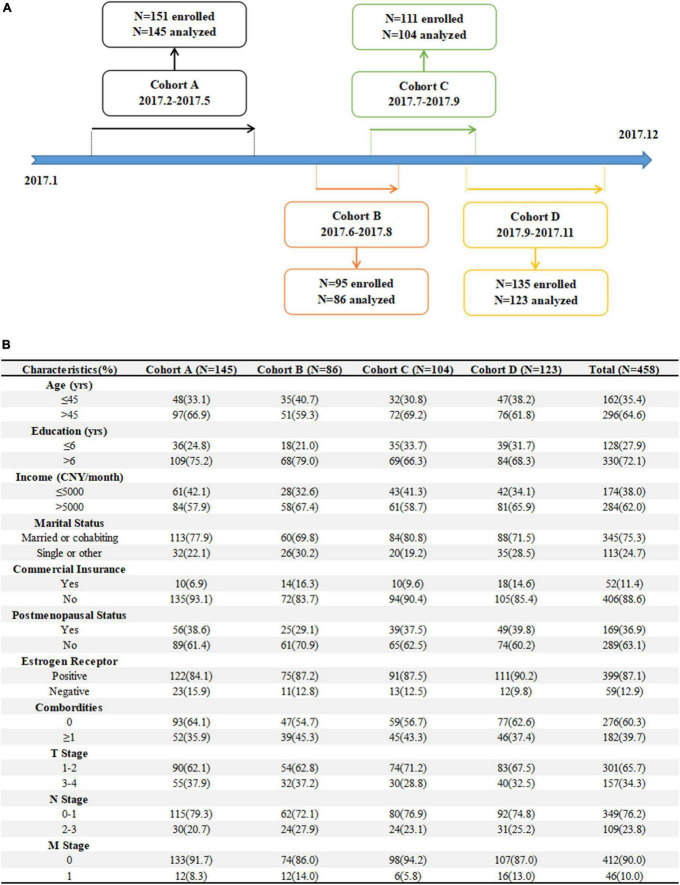
**(A)** The enrollment line **(B)** and demographics for patients.

Four cohorts were developed based on our previous Be Resilient to Breast Cancer (BRBC) (15–18), including: (1) Cohort A, 151 patients were consecutively enrolled from hospital A in Guangzhou between February 2017 and May 2017, (2) Cohort B, 95 patients were consecutively enrolled from hospital B in Foshan between June 2017 and August 2017, (3) Cohort C, 111 patients were consecutively enrolled from hospital C in Jiangmen between July 2017 and September 2017, and (4) Cohort D, 135 patients were consecutively enrolled from hospital D in Zhuhai between September 2017 and November 2017. The enrollment line was described in Figure 1A. The inclusion criteria were: (1) confirmed diagnosis of breast cancer (within 4 weeks), (2) aged >18 years, (3) could communicate in Mandarin or Cantonese fluently, and (4) receiving active treatment. The exclusion criteria were: (1) linguistic or intellectual difficulties, (2) had a currently active Axis I psychiatric disorder, (3) life expectancy less than 12 months, and (4) unwilling to participate in the study. The patients were approached by trained research nurses in the current study and baseline information (T0, i.e., demographics, QoL, resilience, etc.) was collected when their informed consent were obtained. Patients can choose telephone or online interview follow-ups (T1, 6-month) when pencil-and-paper test was not available.

### Ethics approval

The current study was part of BRBC trial and ethic approval number was 2016KYTD08. Informed consent was obtained before formal investigation. Other details about BRBC were described elsewhere (15–18).

### Instruments

#### 10-item Resilience Scale Specific to Cancer (RS-SC-10)

The original RS-SC is a 25-item resilience instrument specific to cancer that has five domains of generic element, benefit finding, support and coping, hope for the future, and meaning for existence (19, 20). RS-SC is rated based on a five-point Likert scale, with higher scores indicating higher resilience levels (score ranges from 25 to 125). In this study, a short-form of 10-item RS-SC (RS-SC-10) was administered (21–23). RS-SC and RS-SC-10 were attached in the Supplementary material.

#### Functional Assessment of Cancer Therapy-Breast (FACT-B)

The Chinese version of FACT-B consists of 37 items and are divided into five domains, including Physical (GP), Social/Family (GS), Emotional (GE), Functional (GF), and Breast Cancer Subscale (BCS) (24). The total score ranges from 0 to 144 with higher scores indicating better functions. In the current study, the changes of FACT-B between T0 and T1 was used as an anchor against resilience. The change group with loss indicated by ≥0.5 SD (standard deviation) of change was defined as Decreased (outcome = 1) and other groups were defined as Non-decreased (outcome = 0).

### Statistical analysis

**FIGURE 2:**
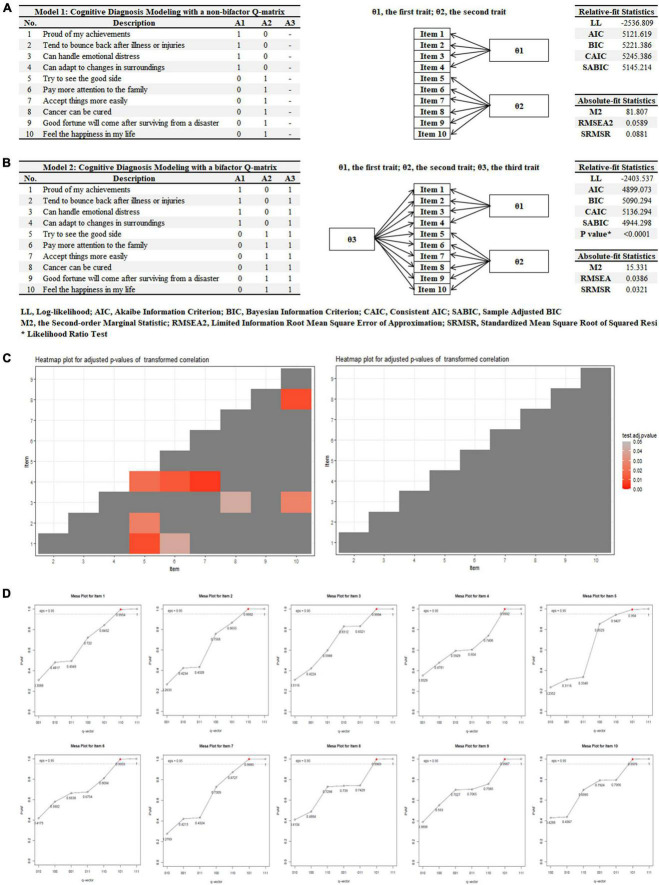
Q-matrix comparison and validation. **(A)** A non-bifactor Q-matrix, **(B)** a bifactor Q-matrix, **(C)** heatmaps for adjusted *p*-values of transformed correlation, and **(D)** mesa plots mapping attributes to items.

First, based on the two-factor structure of RS-SC-10 in our previous research (21–23), two theory-based Q matrix were developed for validating the number of item attributes and detecting the misidentified elements, including a bifactor Q-matrix and a non-bifactor one (Figures 2A, B). A cell with the value of 1 indicates that the corresponding item attribute is captured by the corresponding item.

Second, an unrestricted and saturated model named as Generalized Deterministic Input, Noisy “And” Gate (G-DINA) was utilized in the current study with two different Q matrix to avoid potential model misspecifications. The item response function (IRF) of G-DINA was detailed as below (25):

$$g\left[P\left(\Upsilon_{i\,j}=1|a_{l\,j}^{*}\right)\right]=\delta_{j\,o}+\sum_{k=1}^{K_{j}^{*}}\delta_{j\,k}a_{l\,k}+\sum_{k^{\prime }=k+1}^{K_{j}^{*}}\sum_{k=1}^{K_{j}^{*}-1}\delta_{j\,k\,k^{\prime }}a_{l\,k}a_{l\,k\,k^{\prime }}\ldots +\delta_{j\,12\,\ldots \,K_{j}^{*}}\prod_{k=1}^{K_{j}^{*}}a_{l\,k^{\prime }}$$

where *g*[⋅] represents an identity, logit, or log link function, δ_*j*0_ is the intercept of item *j*, δ*_*jk*_* is the main effect of attribute *k*, δ_*jkk*’_ is the two-way interaction effect of attributes *k* and *k*′, and δ_*j*12.Kj*_ is Kj*-way interaction effect of attributes 1 to Kj*. In addition, a higher-order 2PL model was chosen for the attribute distribution and monotonic constraints was also applied in the G-DINA model.

Third, relative-fit statistics including Akaike Information Criterion (AIC), Bayesian Information Criterion (BIC), Consistent AIC (CAIC), Sample Adjusted BIC (SABIC), as well as a likelihood ratio (LR) test were compared between the two models with different Q matrix (26). In addition, absolute-fit statistics including Second-order Marginal Statistic (M2, less is better), Root Mean Square Error of Approximation (RMSEA, RMSEA < 0.045 is defined as a good fit) and Standardized Mean Square Root of Squared Residuals (SRMSR, SRMSR < 0.05 is defined as a good fit) were also considered (26). Further, a heatmap for residuals between the observed and predicted correlations of item pairs was checked and the optimal G-DINA model was retained for further model evaluation (27).

Fourth, Q-matrix was validated by mesa plots mapping attributes to items (28). The cutoff of Proportion of Variance Accounted For (PVAF) by a particular q-vector was set at 0.95.

Fifth, item parameter including success probabilities of reduced latent classes, guessing and slip parameters, delta parameters, and success probabilities of all latent classes with or without standard errors (SEs) were estimated in the G-DINA model (25).

Sixth, success probabilities of attributes for each person were extracted from the G-DINA model and two models, Model 1: (TNM stage + Total Score) vs. Model 2: (TNM stage + Cognitive Diagnostic Probabilities, were compared based on indicators of AUC, NRI (Net Reclassification Improvement), IDI (Integrated Discrimination Improvement), calibration curves (estimated by Brier score), and Decision Curve Analysis (29, 30). In addition, Clinical Impact Curve were also estimated to evaluate Model 2′ clinical utilizations (31).

At last, Wald test was performed to compare G-DINA model against other reduced model (i.e., DINA, DINO, etc.) at the item level, which could provide better classification results without losing significant fits to models (32). All statistical analyses were performed by GDINA R Package (33).

## Results

### Demographics

Overall, 151, 95, 111, 135 patients were enrolled from four different cohorts. Finally, 145 (96.0%), 86 (90.5%), 104 (93.7%),123 (91.1%) patients completed 6-month follow-up assessments at T1 and a total of 458 patients were analyzed. Compared to those enrolled in the analysis, patients lost to follow-up were reported to have more combordities (*P* = 0.078) and no other significant demographics were identified. The baseline characteristics of the patients were summarized in Figure 1B.

### Q-matrix comparison and validation

Two theory-based Q matrix were developed for validating the number of item attributes and detecting the misidentified elements, including a non-bifactor Q-matrix (Model 1, Figure 2A) and a bifactor one (Model 2, Figure 2B). It demonstrated that Model 2 had better fitting indicators in terms of Relative-fit Statistics (i.e., AIC = 4899.073 vs. 5121.619, BIC = 5090.294 vs. 5221.386, etc.) and Absolute-fit Statistics (i.e., RMSEA = 0.0386 vs. 0.0589, SRMSR = 0.0321 vs. 0.0881, etc.) compared to those based on Model 1. The LR test also confirmed these findings (*X*^2^ = 266.544, df = 24, *P* < 0.0001). In addition, the heatmap for Model 1 demonstrated that residuals between the observed and predicted correlations of item pairs were significant resulting in misfitting (Figure 2C, adjusted *P*-value for transformed correlation = 0.0058). Thus, Model 2 was chosen for further Q-matrix validation. Based on the cutoff of 0.95 of PVAF, mesa plots were provided for mapping attributes to items and no changes should be made to all 10 items. Full details about different q-vectors were described in Figure 2D.

### Item parameter estimation

**FIGURE 3:**
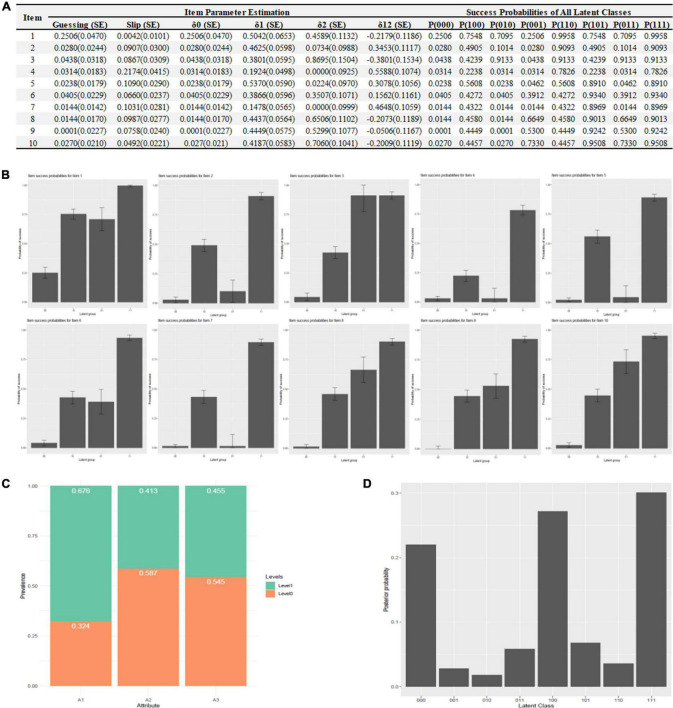
Item parameter estimation. **(A)** Item parameter estimation, **(B)** success probabilities distribution, **(C)** mastery attributes distribution, and **(D)** latent classes distribution.

At the item-level, guessing (*g*) and slip (*s*) parameters with SEs were presented in Figure 3A, indicating the lower (*g*) and upper bound (*1-s*) of success probabilities. In addition, delta parameters (δ) for IRF of G-DINA, and success probabilities of all latent classes (i.e., 000, 100, 010, etc.) were also presented in Figure 3A. The distributions of the success probability across all reduced latent classes in each item were drawn in Figure 3B. It showed that an increase was observed in the success probability as a patient mastered more attributes in most items (i.e., item 8, 9, 10, etc.) indicating a perfect fitting. At the test-level, 67.6, 41.3, 45.6% of patients successfully mastered “Generic Elements (A1),” “Shift-Persist (A2),” “General Factor (A3)” which were described in Figure 3C. In addition, the most proportions of latent classes were “111,” “100,” and “000,” accounting for 30.0, 27.2, 22.0% of the patients, respectively. Other details about latent classes could be identified in Figure 3D. The accuracy at test level was 0.8377 and were 0.9528 (A1), 0.9042 (A2), and 0.9155 (A3) at attribute level, respectively.

### The comparison of different prediction models (total score vs. cognitive diagnostic probabilities)

**FIGURE 4:**
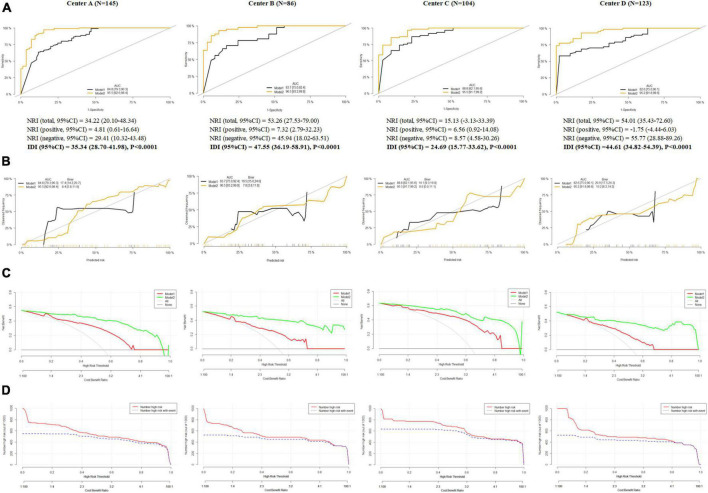
The comparison of different prediction models (total score vs. cognitive diagnostic probabilities). **(A)** Area under curve, net reclassification improvement and integrated discrimination improvement in Model 1 and Model 2, **(B)** calibration curves in Model 1 and Model 2, **(C)** decision curve analysis in Model 1 and Model 2, and **(D)** clinical impact curves in Model 1 and Model 2.

Two models were developed to construct the prediction model for Decreased QoL outcome, including: Model 1: TNM stage + Total Score and Model 2: TNM stage + Cognitive Diagnostic Probabilities. Compared with Model 1, AUC in Model 2 increased from 82.6–88.8 to 95.2–96.5% in four cohorts (all *P* < 0.001, Figure 4A). The NRI in Model 2 ranged from 15.13 to 54.01% and IDI ranged from 24.69 to 47.55% (all *P* < 0.0001, Figure 4A). In addition, Brier scores in Model 2 ranged from 7.8 to 10.2, which were significantly less than those in Model 1 (ranged from 14.1 to 20.9, Figure 4B). DCA indicated that Model 2 showed higher net benefits compared with Model 1 (Figure 4C) in different cohorts. Thus, Model 2 had better predictable ability to Decreased QoL outcome than Model 1 and CICs about Model 2′ clinical utilization in different cohorts were detailed in Figure 4D.

### Item-level model comparison

**FIGURE 5:**
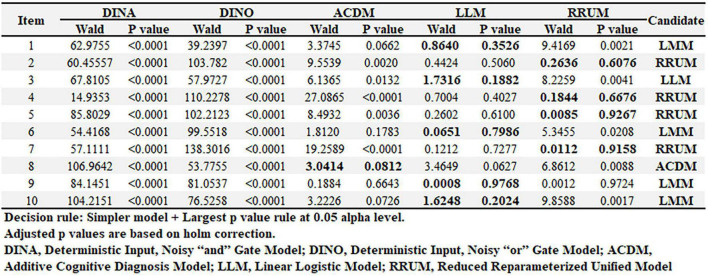
Item-level model comparison.

Wald test indicated that the DINA model could be replaced by several reduced cognitive diagnostic models. For example, in item 1, 3, and 6, Linear Logistic Model (LLM) could provide better classification results without losing significant fits to models. Other information about Additive Cognitive Diagnosis Model (ACDM) and Reduced Reparameterized Unified Model (RRUM) could be recognized in Figure 5.

## Discussion

This is the first study to measure resilience by CDMs and its prediction of 6-month Quality of Life (QoL) in breast cancer, though CDMs have been performed to analyze PROs in pathological gambler, schizophrenia, internet addition, etc (12, 13, 34). The findings demonstrated in this manuscript confirmed the usefulness of CDMs as a sophisticated method for jointly performing resilience assessment and investigating theory-based structural facets of resilience traits. With the additional item-level information from G-DINA model, the prediction ability of resilience to 6-month QoL was significantly enhanced. Thus, the underlying structure of resilience traits or other psychological indicators (i.e., depression, anxiety, etc.) should also be considered in future PROs research. Furthermore, G-DINA-based RS-SC-10 could reduce the burden of psychologist/psychiatrist when large patients are screened or monitored.

However, two important issues in the application of CDMs should be discussed here. First, a theoretical framework or Q matrix should be set for CDMs before the formal analysis and the results will have limited diagnostic information if the theoretical framework is not relevant to the diagnosis. For example, based on our previous study, two theory-based Q matrix were developed for validating the number of item attributes and detecting the misidentified elements, including a non-bifactor Q-matrix (Model 1, Figure 2A) and a bifactor one (Model 2, Figure 2B). If both Q matrix are biased, misguided Q-matrices will lead to meaningless results with inflated slip and guess parameters, high RMSE and low Monte Carlo *p*-values. However, there exists no golden standard/definition for resilience and whether resilience should be conceptualized as a trait or a state is also debated (35–37). Therefore, we should be cautious about the exploratory Q matrix-based findings derived from the current study which should be replicated and validated in future research. Also, Q-matrix discovery techniques have been developed to handle with the issue about unknown Q-matrix. For example, a Q-matrix discovery method has been developed to allows for uncertainty in instrument development process by using experts’ comments in a Bayesian algorithm (38).

Second, the cognitive diagnostic probabilities (CDPs) should be validated by external indicators. In the current study, CDPs of resilience were anchored against 6-month QoL based on a prospective cohort design. However, researchers might prefer that external validation come not only from PRO instruments but also from diagnoses elicited from trained physicians. More objective indicators, i.e., cortisol, C-reactive protein, systolic pressure, etc., can be incorporated into the CDMs research, which will give more confidence to the interpretation of results (39, 40). In addition, we should be noted that CDMs take complex interactions among latent variables into consideration which allows greater flexibility than most IRT models in modeling item responses. However, this also results in complex model with too many parameters. In the current study, several reduced models have been recommended for replacing G-DINA (Figure 5) and these models can be tried in the future research to obtain more stable parameter estimates. At last, if a long item instrument with many attributes is administered in a large sample, computerized adaptive testing (CAT) can be considered to reduce the test time without a loss of measurement precision. Some research on combining CDMs and CAT has been explored in the field of psychometrics and CDMs-CAT is promising in the field of medicine (41, 42).

## Limitation

There are several limitations should be considered. First, many parameters are involved in G-DINA and the sample size in the current study may not be large enough resulting in potential biased estimation of Wald test for model selection or DIF detection (43). Larger sample should be considered in future research to get a robust estimated covariance matrix. Second, our sample was collected from an extremely narrow Chinese population with breast cancer and the generalizability of these findings should be further validated in other populations (i.e., lung cancer, colon-rectal cancer, etc.). In addition, CDMs allow for measurement of differences in attribute patterns cross-culturally which can help explain how cultural differences are manifested in the patterns of attributes mastered (44, 45). Third, RS-SC-10 has been validated in family caregivers (i.e., parents of children with cancer) as well as patients with different cancer types, and CDMs might also have great potentials in these vulnerable populations (46–50). More CDM-based evaluation and intervention for these vulnerable groups are urgently warranted. At last, although various CDMs have been developed to detect mastery and non-mastery of multiple fine-grained skills or attributes (i.e., DINO, DINA, ACDM, RRUM, etc.), most researchers in the field of medicine are still not familiar with CDMs due to their novelty as well as complex statistics. There exist several software programs available to estimate CDMs, for example, several R packages are available for the CDMs analyses. However, syntax preparation of these programs requires researchers’ substantial effort and more easy-use software should be developed to facilitate the application of CDMs.

## Conclusion

Cognitive diagnostic probabilities of resilience contribute to a more accurate prediction of 6-month QoL above conventional total score. CDMs could help optimize Patient Reported Outcomes (PROs) measurement in breast cancer.

## Data availability statement

The raw data supporting the conclusions of this article will be made available by the authors, without undue reservation.

## Ethics statement

The studies involving human participants were reviewed and approved by Guangzhou University of Chinese Medicine. The patients/participants provided their written informed consent to participate in this study.

## Author contributions

ML and PC: investigation, formal analysis, supervision, and writing—original draft. MK and AM: methodology and writing—review and editing. YT: investigation and visualization. GH and ZS: investigation. YY: data curation and software. ZY: conceptualization, funding acquisition, project administration, resources, and writing—review and editing. All authors contributed to the article and approved the submitted version.
