# Supplementary material for: Measuring resilience by cognitive diagnosis models and its prediction of 6-month quality of life in Be Resilient to Breast Cancer (BRBC)
Source: Front Psychiatry. 2023 Feb 16;14:1102258. doi: 10.3389/fpsyt.2023.1102258 (PMC9978149; doi:10.3389/fpsyt.2023.1102258)
Supplement: Supplementary file 1 [file Table_1.DOCX]

**25-item Resilience Scale Specific to Cancer (RS-SC-25)**

**Instructions:** Please read the items below and indicate how often you agree with these statements over the last 4 weeks. If some specific situations did not occur, imagine about how you would feel if it had happened. There are no right or wrong answers and please circle on the number which most closely described your feelings.

| **Item** | **Content** | **Never** | **Seldom** | **Sometimes** | **Often** | **Always** |
| --- | --- | --- | --- | --- | --- | --- |
| 1 | Have a clear goal in my life | 1 | 2 | 3 | 4 | 5 |
| 2 | Proud of my achievements | 1 | 2 | 3 | 4 | 5 |
| 3 | Tend to bounce back after illness or injuries | 1 | 2 | 3 | 4 | 5 |
| 4 | Can handle emotional distress | 1 | 2 | 3 | 4 | 5 |
| 5 | Can adapt to changes in my surroundings | 1 | 2 | 3 | 4 | 5 |
| 6 | Able to control my life | 1 | 2 | 3 | 4 | 5 |
|  | **When you are faced by cancer,** |  |  |  |  |  |
| 7 | Try to see the good side | 1 | 2 | 3 | 4 | 5 |
| 8 | Adapt to things cannot be changed | 1 | 2 | 3 | 4 | 5 |
| 9 | Pay more attention to family | 1 | 2 | 3 | 4 | 5 |
| 10 | Accept things more easily | 1 | 2 | 3 | 4 | 5 |
| 11 | Show more empathy for others | 1 | 2 | 3 | 4 | 5 |
| 12 | Able to handle side effects of treatment | 1 | 2 | 3 | 4 | 5 |
| 13 | Family will support my decisions | 1 | 2 | 3 | 4 | 5 |
| 14 | Establish good relationship with other patients, nurses and physicians | 1 | 2 | 3 | 4 | 5 |
| 15 | Can relieve emotional distress in my own way | 1 | 2 | 3 | 4 | 5 |
| 16 | Keep doing my hobbies | 1 | 2 | 3 | 4 | 5 |
|  | **When you are faced by cancer,** |  |  |  |  |  |
| 17 | Cancer can be cured | 1 | 2 | 3 | 4 | 5 |
| 18 | Still have many plans for my life | 1 | 2 | 3 | 4 | 5 |
| 19 | I believe that good fortune will come after surviving a disaster | 1 | 2 | 3 | 4 | 5 |
| 20 | Praise for good deeds | 1 | 2 | 3 | 4 | 5 |
| 21 | Have a strong sense of purpose for life | 1 | 2 | 3 | 4 | 5 |
| 22 | Feel the happiness in my life | 1 | 2 | 3 | 4 | 5 |
| 23 | Everyone should take responsibility for their own life | 1 | 2 | 3 | 4 | 5 |
| 24 | Everyone should pursue something good during life | 1 | 2 | 3 | 4 | 5 |
| 25 | I can accept that all people are mortal | 1 | 2 | 3 | 4 | 5 |

**10-item Resilience Scale Specific for Cancer (RS-SC-10)**

**Instructions:** Please read the items below and indicate how often you agree with these statements over the last 4 weeks. If some specific situations did not occur, imagine about how you would feel if it had happened. There are no right or wrong answers and please circle on the number which most closely described your feelings.

| **Item** | **Content** | **Never** | **Seldom** | **Sometimes** | **Often** | **Always** |
| --- | --- | --- | --- | --- | --- | --- |
| 1 | Proud of my achievements | 1 | 2 | 3 | 4 | 5 |
| 2 | Tend to bounce back after illness or injuries | 1 | 2 | 3 | 4 | 5 |
| 3 | Can handle emotional distress | 1 | 2 | 3 | 4 | 5 |
| 4 | Can adapt to changes in my surroundings | 1 | 2 | 3 | 4 | 5 |
|  | **When you are faced by cancer,** |  |  |  |  |  |
| 5 | Try to see the good side | 1 | 2 | 3 | 4 | 5 |
| 6 | Pay more attention to family | 1 | 2 | 3 | 4 | 5 |
| 7 | Accept things more easily | 1 | 2 | 3 | 4 | 5 |
| 8 | Cancer can be cured | 1 | 2 | 3 | 4 | 5 |
| 9 | I believe that good fortune will come after surviving a disaster | 1 | 2 | 3 | 4 | 5 |
| 10 | Feel the happiness in my life | 1 | 2 | 3 | 4 | 5 |
